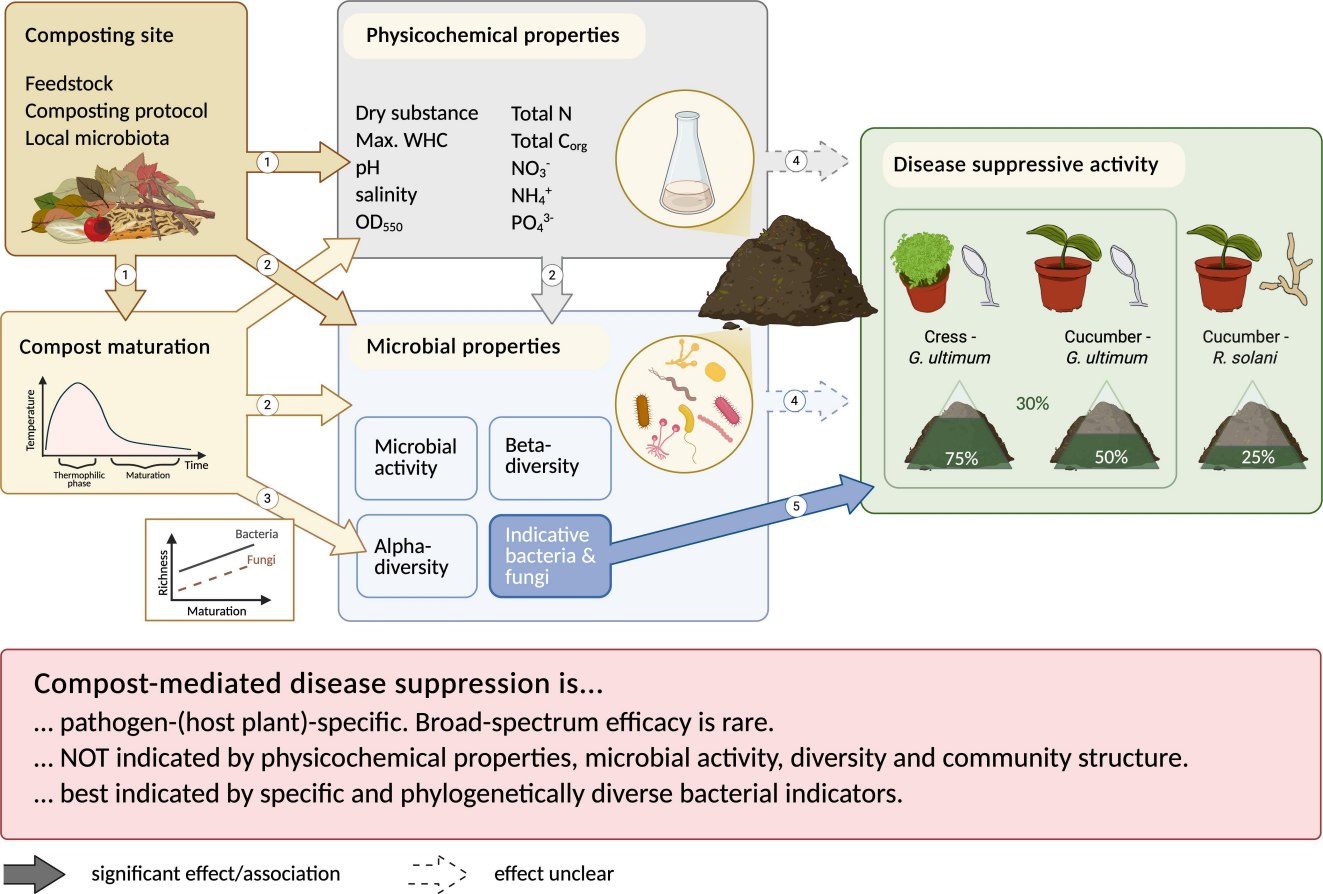# Articles of Significant Interest in This Issue

**DOI:** 10.1128/aem.02460-25

**Published:** 2025-12-23

## PLANT-MICROBE CONVERSATIONS VIA VESICLES

This minireview by Zannis-Peyrot et al. (e01766-25) discusses the many roles of phytobacterial extracellular vesicles (EVs) in plant-bacterium interactions, from colonization to interkingdom communication.


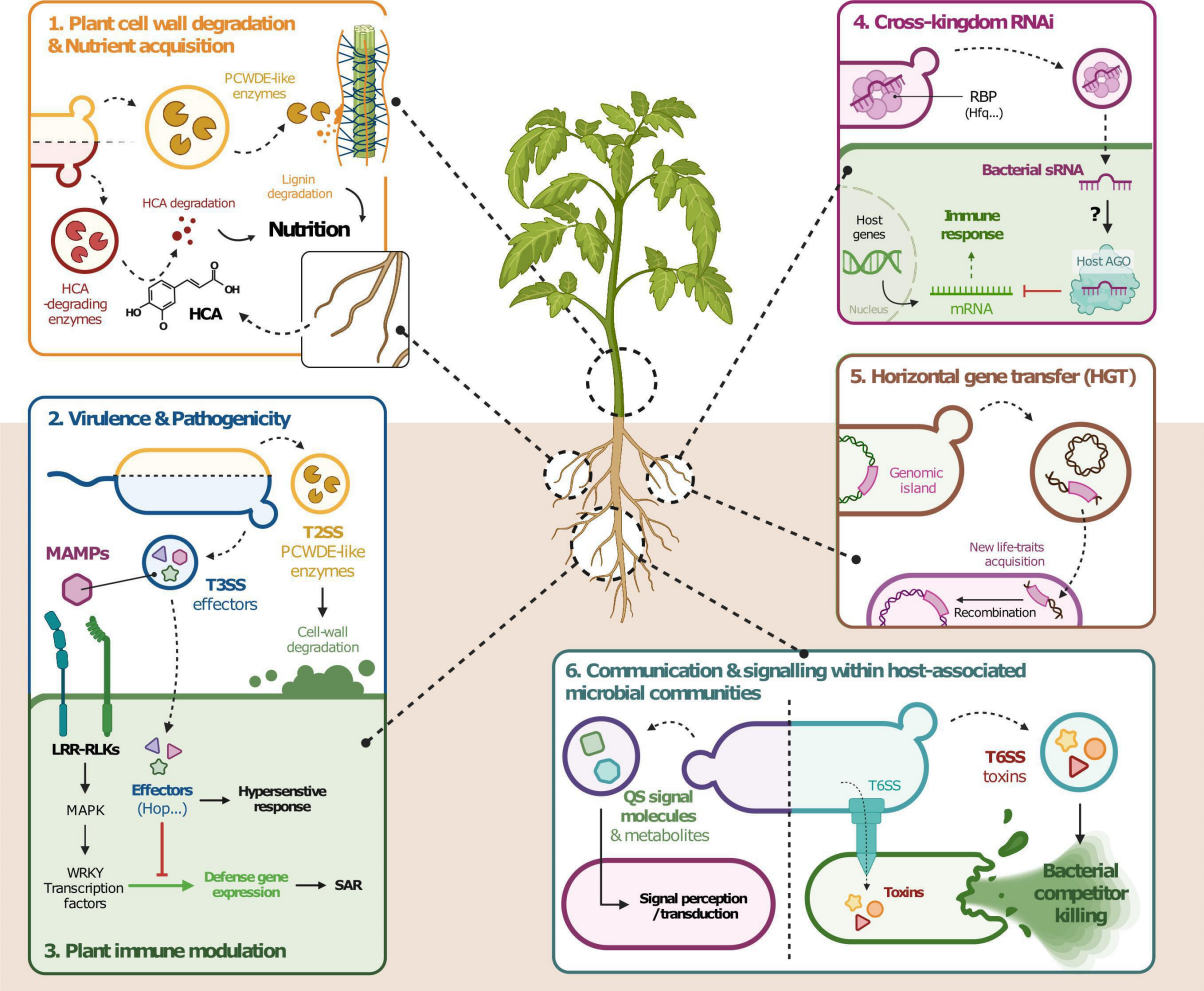


## A TASTE FOR LONG ALKANES

Zhang et al. (e02124-25) describe a bacterial strain that can degrade medium- and long-chain (C16–C36) *n*-alkanes and branched alkanes (pristane), providing a promising option for oily sludge bioremediation. 


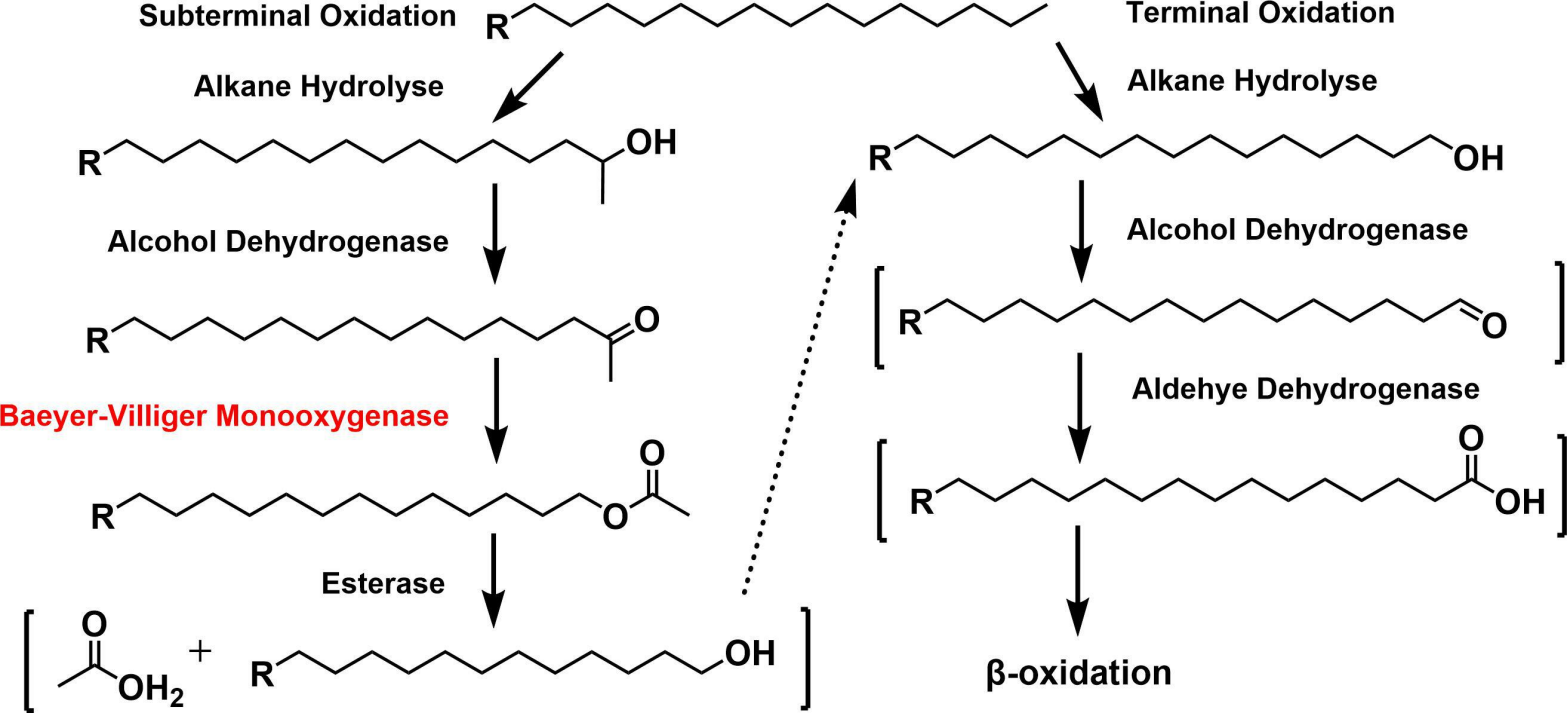


## DANGEROUS LIAISONS BETWEEN TWO METALS

Nickel (Ni) and copper (Cu) are common co-contaminants. Darwiche et al. (e01627-25) show that, while relatively harmless individually, these metals disrupt iron-sulfur (Fe-S) clusters when combined. The ubiquity and essentiality of Fe-S clusters across microbial taxa suggest that these two common co-contaminants may be toxic to diverse microorganisms.


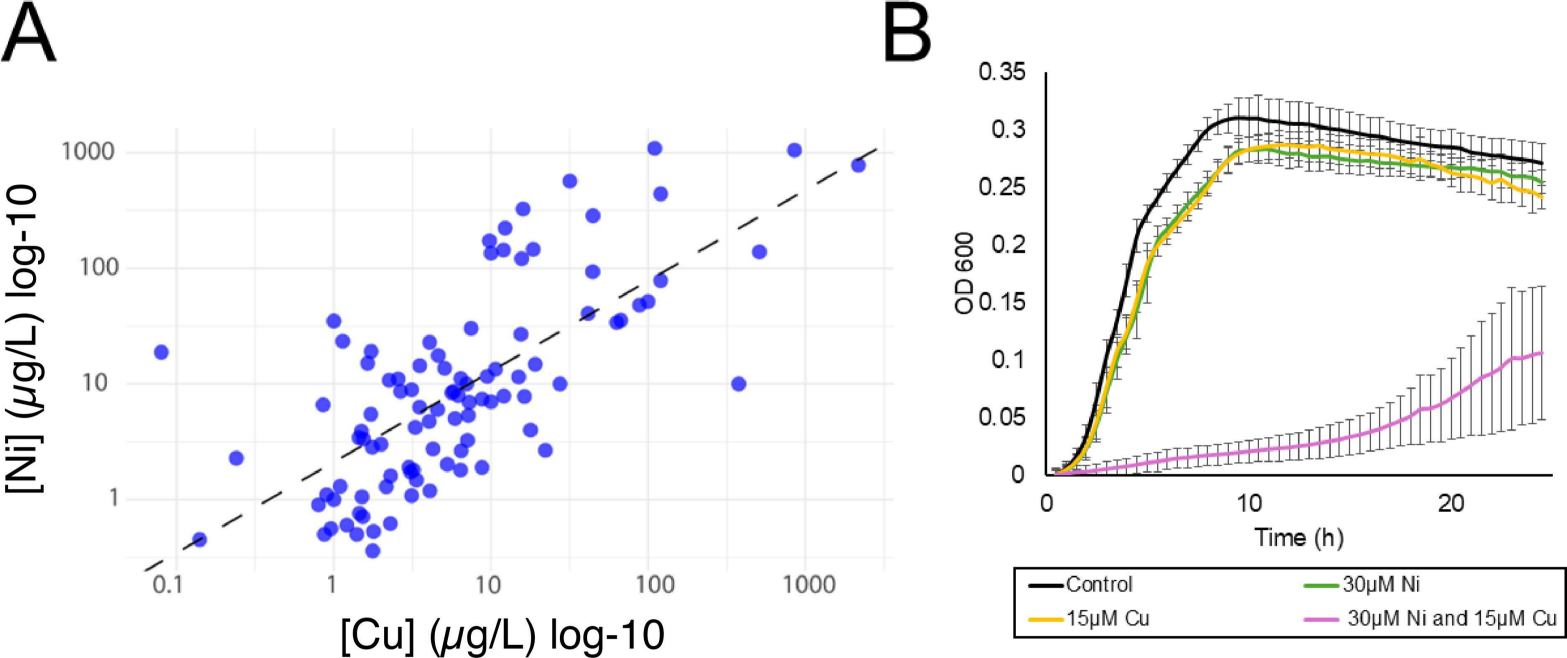


## BISPHENOL F DEGRADATION PATHWAY REVEALED

Bisphenol F (BPF) is an emerging environmental pollutant with endocrine-disrupting effects that is widespread in surface water and wastewater systems. Chen et al. (e01830-25) describe the genetic basis that defines the enzymatic steps for the degradation of this pollutant.


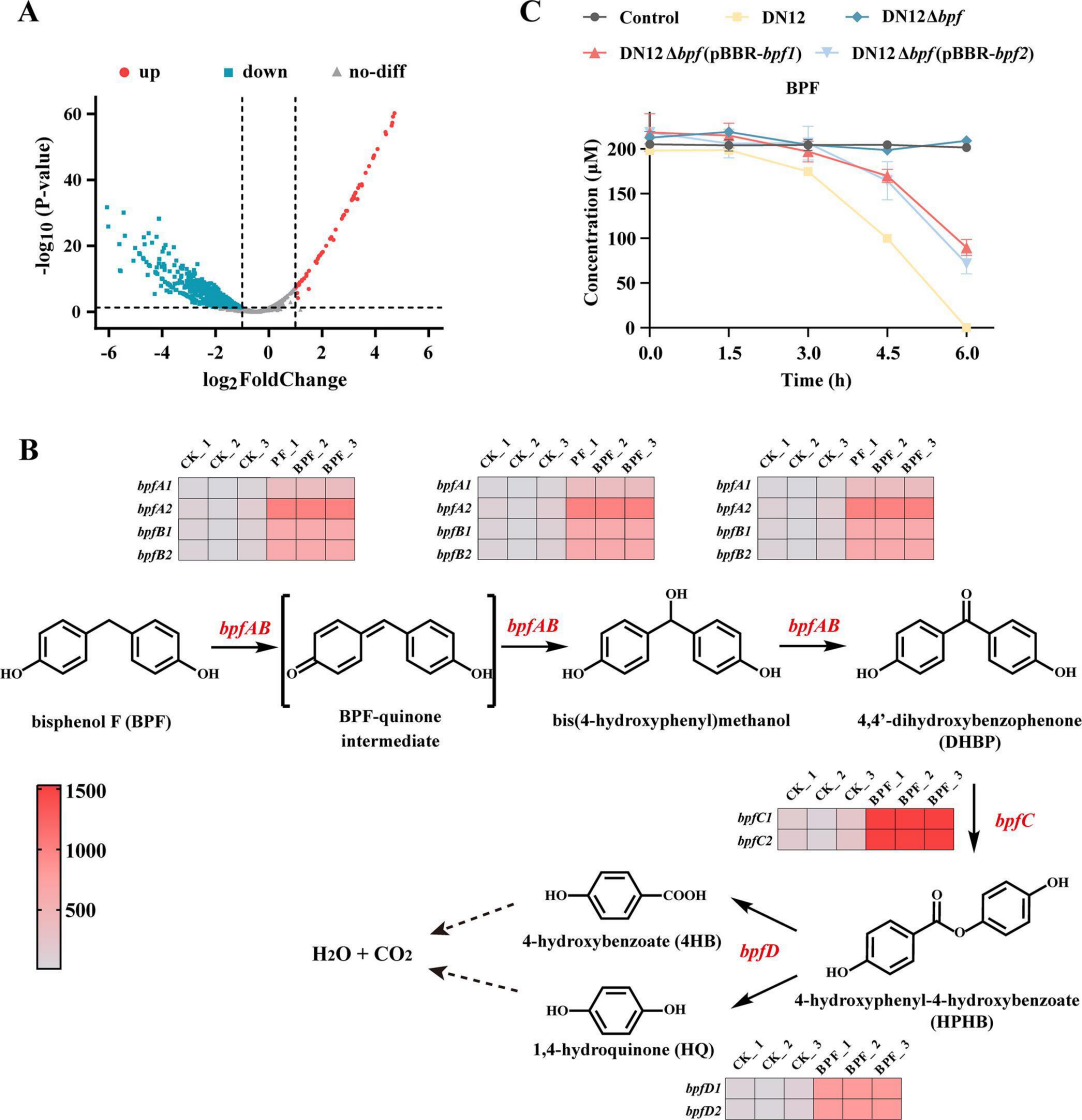


## METHANE AND SULFATE IN OIL HARMONY

Beilig et al. (e00141-25) show that methanogenesis and sulfate reduction can coexist in offshore oil reservoirs, challenging established paradigms of microbial competition and metabolic exclusivity among these two groups.


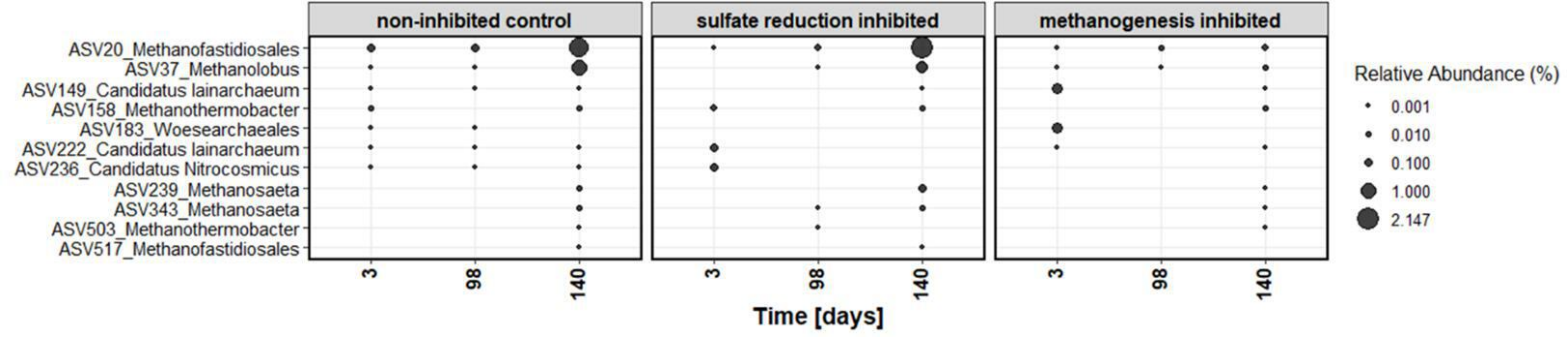
 

## OVERCOMING BARRIERS TO PHAGE INFECTION OF FOODBORNE PATHOGENS

Some strains of *Salmonella* resist infection by phage F01, a widely used biocontrol agent in the food industry. Bosma et al. (e01384-25) describe the prophage-encoded defense protein behind the infection barrier.


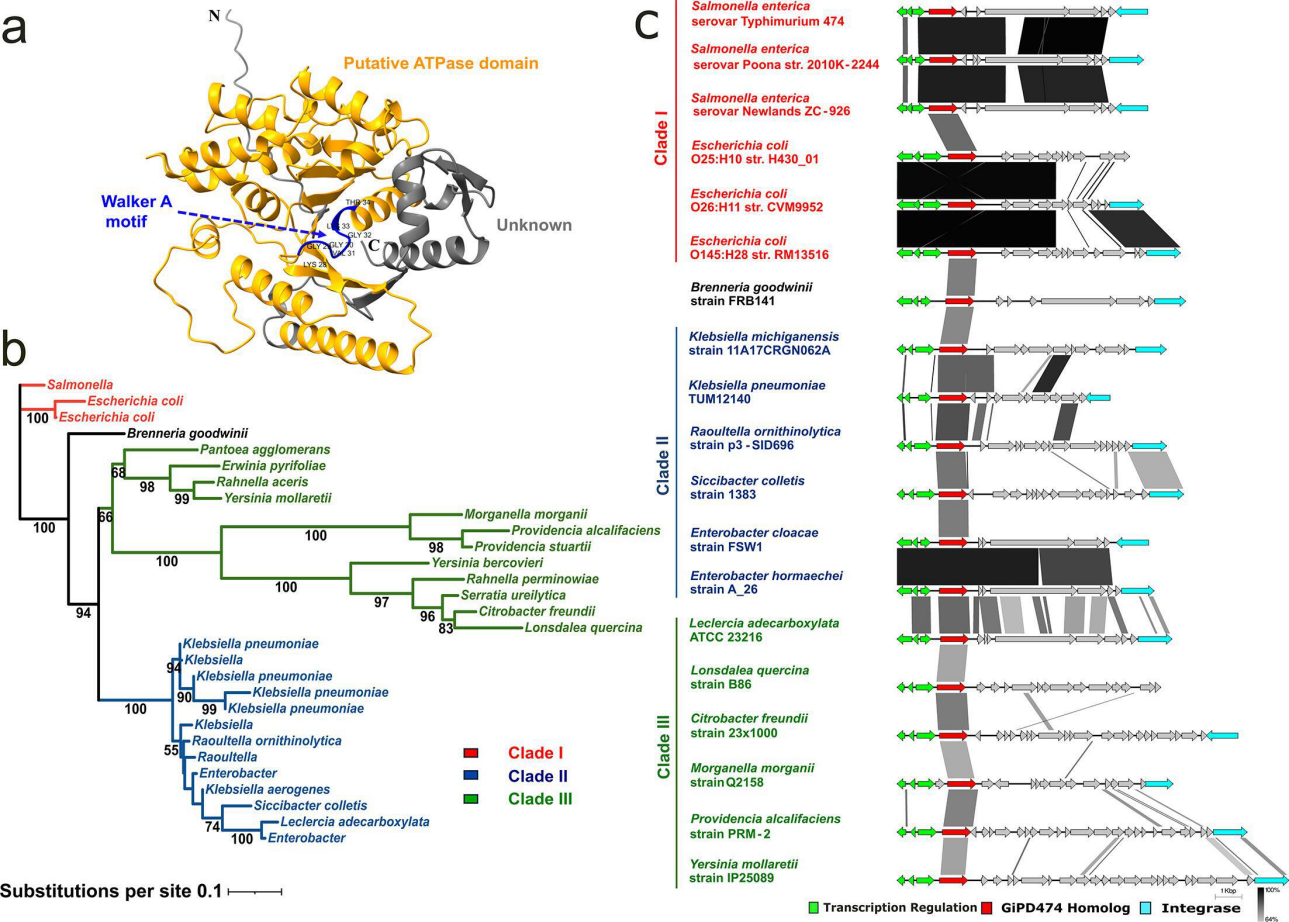


## SUPPRESSIVE COMPOSTS, DISSECTED

This commentary by Bonanomi and Idbella (e02019-25) discusses recent findings of biomarkers of compost suppressiveness and knowledge gaps towards predicting compost capacity to suppress pathogens across various systems.